# Comorbidity Between DSM–IV Alcohol and Specific Drug Use Disorders in the United States: Results From the National Epidemiologic Survey on Alcohol and Related Conditions

**Published:** 2006

**Authors:** Frederick S. Stinson, Bridget F. Grant, Deborah A. Dawson, W. June Ruan, Boji Huang, Tulshi Saha

**Affiliations:** Laboratory of Epidemiology and Biometry, Division of Intramural Clinical and Biological Research, National Institute on Alcohol Abuse and Alcoholism, National Institutes of Health, Department of Health and Human Services, Bethesda, MD

**Keywords:** DSM–IV alcohol abuse, DSM–IV alcohol dependence, Epidemiology, Comorbidity

## Abstract

**Background:**

To date, there have been no published data on 12-month comorbidity of DSM–IV alcohol and drug use disorders in the general U.S. population. The purposes of the present study were to examine the prevalence and comorbidity of alcohol and specific drug use disorders, and to identify sociodemographic and psychopathologic correlates and treatment-seeking among three groups of respondents: (1) those with alcohol use disorders only; (2) those with drug use disorders only; (3) those with comorbid alcohol and drug use disorders.

**Methods:**

Information on 12-month alcohol and specific drug use disorders in the United States was derived from face-to-face interviews in the National Institute on Alcohol Abuse and Alcoholism’s (NIAAA) 2001–2002 National Epidemiologic Survey on Alcohol and Related Conditions (NESARC) (n = 43,093).

**Results:**

Prevalences were 7.35 percent for alcohol use disorders only, 0.90 percent for drug use disorders only, and 1.10 percent for comorbid alcohol and drug use disorders. Sociodemographic and psychopathologic correlates of these three groups were quite different, with the drug use disorder and comorbid groups significantly more likely to be young, male, never married, and of lower socioeconomic status than the alcohol use disorder only group. Associations between current alcohol use disorders and 25 specific drug use disorders were generally positive and statistically significant. The 12-month prevalence of treatment-seeking significantly increased, from 6.06 percent for those with an alcohol use disorder only to 15.63 percent for those with a drug use disorder only, and to 21.76 percent for those with comorbid alcohol and drug use disorders.

**Conclusions:**

This study provides detailed data on the homotypic comorbidity of alcohol use disorders and 25 different drug use disorders and confirms the high levels of association seen in previous studies based on lifetime measures. Implications of this study are discussed in terms of integrating alcohol and drug treatment services and refining prevention and intervention efforts.

## Introduction

Over the past 20 years, there has been a growing interest in the co-occurrence or comorbidity of psychiatric disorders. In general, comorbidity refers to the co-occurrence or overlap of two or more psychiatric disorders. The term “dual diagnosis” refers more specifically to the co-occurrence of substance (alcohol or drug) use disorders and other psychiatric disorders. Recently, both terms have been combined to produce definitions of homotypic comorbidity, the co-occurrence of disorders, within a diagnostic grouping (e.g., sedative dependence and alcohol use disorders), and heterotypic comorbidity, the co-occurrence of two disorders from different diagnostic groupings (e.g., alcohol use disorders and major depression) (Angold et al., 1999). Despite the enormous literature on heterotypic comorbidity and a substantial body of research on homotypic comorbidity among nonsubstance use disorders in recent years, relatively little is known about homotypic comorbidity between alcohol and drug use disorders. Moreover, there exists a paucity of research on the impact of this form of homotypic comorbidity on alcohol and drug treatment-seeking.

Although alcohol and illicit drug abuse are among the top 10 major risk factors in global burden of mortality and morbidity (Murray and Lopez, 1996), until recently only a few national surveys have been conducted worldwide that have assessed homotypic comorbidity of alcohol and drug use disorders. Recognizing the need for prevalence and comorbidity data on alcohol and drug use disorders, the World Health Organization (WHO) (2000) established the World Mental Health Consortium in 1998 to address, in part, such limitations. During 2000–2002, epidemiological surveys were conducted worldwide in 14 countries. However, studies conducted in Belgium, France, Germany, Italy, the Netherlands, Spain, and Ukraine collected data on alcohol, but not drug use disorders (World Health Organization World Mental Health Consortium, 2004), rendering the study of homotypic alcohol and drug use disorder comorbidity impossible. Fortunately, WHO Consortium surveys conducted in Colombia, Mexico, Lebanon, Nigeria, Japan, and the People’s Republic of China did assess alcohol and drug use disorders and preliminary data from these surveys are forthcoming. Once available, cross-cultural comparisons with the United States data presented here will be possible for the first time.

To date, only five large epidemiologic studies of the general population have examined the homotypic comorbidity of alcohol and drug use disorders worldwide. The first was the Epidemiologic Catchment Area (ECA) Survey, conducted in five U.S. sites in the early 1980s (Regier et al., 1990). The second was the 1990–1992 National Comorbidity Survey (NCS), a nationally representative sample of the United States (Kessler et al., 1997). The third was the National Longitudinal Alcohol Epidemiologic Survey (NLAES), also a nationally representative sample of the United States (Grant, 1992). The remaining two epidemiologic surveys were conducted in other countries: the 1990 Mental Health Supplement of the Ontario Canada Health Survey (MHS–OHS) (Ross, 1995); and the 1997 Australian National Survey of Mental Health and Well-Being (NSMHWB) (Hall et al., 1999).

Data from these five large-scale surveys of the general population were used to examine: (1) the conditional probability of having an alcohol use disorder among those with a drug use disorder, (2) the conditional probability of having a drug use disorder among those with an alcohol use disorder, and/or (3) the associations between alcohol and drug use disorders (Agosti et al., 2002; Burns and Teesson, 2002; Degenhardt et al., 2001; Grant and Pickering, 1996; Helzer and Pryzbeck, 1988; Kessler et al., 1997; Regier et al., 1990; Ross, 1995). Taken together, all of these studies showed that conditional probabilities of having an alcohol use disorder among those with a drug use disorder were significantly greater than among those without a drug use disorder and vice versa. With few exceptions, associations between alcohol and drug use disorders also were positive and significant.

The literature focusing on the impact of homotypic comorbidity of alcohol and drug use disorders on alcohol and drug treatment-seeking is sparse and substantially smaller than the corresponding body of research focusing on heterotypic comorbidity. One of the reasons for this is that ECA, NCS, MHS–OHS, and NSMHWB did not collect treatment utilization information that was diagnostic specific. That is, global information was collected on the use of services for alcohol, drug, and/or mental disorders (Agosti et al., 2002; Burns and Teesson, 2002; Helzer and Pryzbeck, 1988; Kessler et al., 1997; Regier et al., 1990; Ross, 1995; Wu et al., 1999). In contrast, Grant and Pickering (1996) used the NLAES data to examine the influence of current (12-month) alcohol and drug use disorders on current (12-month) alcohol and drug help-seeking. Help-seeking for alcohol and drug problems was ascertained separately in NLAES, and respondents were asked to separately indicate whether they had sought treatment at 13 different treatment sources for alcohol problems and 14 different treatment sources for drug problems. In that study, the presence of a current comorbid drug use disorder among individuals with a current alcohol use disorder doubled the rate of seeking alcohol treatment compared to those without comorbid alcohol use disorders, but a concomitant increase was not observed among individuals with a current drug use disorder who had a current alcohol use disorder.

These studies all contributed much valuable information. However, they leave important questions unanswered about the current homotypic comorbidity of alcohol and drug use disorders and the influence of that comorbidity on alcohol and drug treatment-seeking. First, all but two (Burns and Teesson, 2002; Degenhardt et al., 2001) of the studies reviewed reported prevalences and homotypic comorbidity on a life-time, rather than current (i.e., 12-month) basis. The two Australian studies that used 12-month rates did not provide estimates for all specific drug use disorders, collapsing abuse and dependence categories and/or combining specific drug use disorder categories into global measures of abuse/dependence. With the exception of the study by Grant and Pickering (1996), studies focusing on lifetime homotypic comorbidity also were limited by reporting rates for an aggregated measure of drug use disorders. Perhaps what is most significant is the lack of information on the sociodemographic and psychopathologic correlates of current alcohol use disorders, drug use disorders, and comorbid alcohol and drug use disorders. Information on current comorbidity is important, since it minimizes recall bias typical of lifetime data and reflects alcohol and drug use disorders that cooccur in the last 12 months as opposed to over the lifetime. Further, only NLAES and NSMHWB reported homotypic comorbidity data using the most recent diagnostic criteria, the *Diagnostic and Statistical Manual of Mental Disorders, Fourth Edition* (DSM–IV) (American Psychiatric Association, 1994). As previously noted, none of the earlier surveys except NLAES reported alcohol- and drug-specific treatment-seeking.

Accordingly, the present study was designed to address these limitations using the 2001–2002 National Institute on Alcohol Abuse and Alcoholism’s (NIAAA) National Epidemiologic Survey on Alcohol and Related Conditions (NESARC). NESARC, a large (*n* = 43,093) representative sample of the U.S. population, allows for the examination of the current prevalences and associations between alcohol and specific drug use disorders and the impact of their comorbidity on alcohol and drug treatment-seeking. Obtaining accurate information on current alcohol and specific drug use disorders is important, since etiology and treatment implications of disorders within broad categories often differ considerably. This study also identifies high-risk subgroups of the population defined by sociodemographic and psychopathologic correlates among individuals with alcohol use disorders, with drug use disorders, and with comorbid alcohol and drug use disorders for the purpose of refining prevention and intervention efforts. Further, an update of the earlier studies reviewed here is critical since recent comparisons between NLAES and NESARC have shown significant increases in alcohol use disorders (Grant et al., 2004c) and drug use disorders (Compton et al., 2004) over the last decade of the 20th century.

## Methods

### NESARC Sample

The 2001–2002 NESARC is a representative sample of the United States conducted by NIAAA that has been described in detail elsewhere (Grant et al., 2003b). The target population of NESARC was the civilian noninstitutionalized population, age 18 and older, residing in the United States and the District of Columbia, including Alaska and Hawaii. The sample included persons living in households, the military living off base, and the following group quarters: boarding houses, rooming houses, nontransient hotels and motels, shelters, facilities for housing workers, college quarters, and group homes. Face-to-face personal interviews were conducted with 43,093 respondents. The sampling frame response rate was 99 percent, the household response rate was 89 percent, and the person response rate was 93 percent, yielding an overall survey response rate of 81 percent.

Oversampling of Blacks and Hispanics was accomplished at the design phase of the survey. Oversampling increased the proportion of Hispanic and Black households to approximately 20 percent each of the total sample. For each housing unit, one person was selected randomly from a roster of persons living in the household. At this stage in the survey design, young adults (ages 18–24) were oversampled at a rate of 2.25:1.00.

The NESARC data were weighted to reflect the probabilities of selection of primary sampling units (PSUs) within strata and for the selection of housing units within the sample PSUs. The data were also weighted to: (1) account for the selection of one sample person from each household; (2) account for oversampling of young adults; (3) adjust for nonresponse at the household level and person level; (4) reduce the variance arising from selecting two PSUs to represent an entire stratum. The weighted data were then adjusted to be representative of the United States civilian noninstitutionalized population for a variety of socioeconomic variables including region, age, sex, race, and ethnicity using the 2000 Decennial Census of Population and Housing.

### Interviewer Training and Field Quality Control

Approximately 1,800 experienced lay interviewers from the United States Bureau of the Census administered NESARC using laptop computer-assisted software that included built-in skip, logic, and consistency checks. On average, the interviewers had 5 years’ experience working on Census and other healthrelated national surveys. All NESARC interviewers completed a 5-day self-study at home and participated in a standardized 5-day in-class training session at one of the Bureau’s 12 regional offices. NESARC training supervisors from each regional office also were required to complete the home study and to attend a centralized training session prior to fielding of the survey, where they completed the in-class training under the direction of NIAAA sponsors and Census Field and Demographics Survey Division Headquarters staff.

Regional supervisors recontacted a random 10 percent of all respondents for quality control purposes. In these quality control interviews, a series of questions were reasked to verify that respondents had received the entire interview and that the questionnaire had been administered properly. There was no case in which it was determined that the interview had been conducted in any manner that was inconsistent with the interviewer’s extensive training. In addition, 2,657 respondents were randomly selected to participate in a reinterview study after completion of their NESARC interview. Each respondent was readministered one to three sections of the survey assessment instrument. These interviews not only served as an additional check on survey data quality but also formed the basis of a test–retest reliability study of new modules of the survey instrument (Grant et al., 2003a).

### Alcohol and Drug Use Disorder Assessment

Diagnoses presented in this report were made by NIAAA’s AUDADIS–IV (Grant et al., 2001), a state-of-the-art structured diagnostic interview designed to be used by lay interviewers. AUDADIS–IV included an extensive list of symptom questions that separately operationalized DSM–IV criteria for alcohol and drug abuse and dependence for 10 classes of drugs: sedatives, tranquilizers, opiates (other than heroin or methadone), stimulants, hallucinogens, cannabis, cocaine (including crack cocaine), inhalants/solvents, heroin, and other drugs. Consistent with DSM–IV, current (last 12 months) dependence diagnoses required the respondent to satisfy at least three of the seven DSM–IV criteria for dependence during the last year. For those DSM–IV substance use disorders for which withdrawal is a dependence criterion (i.e., alcohol, sedatives, tranquilizers, opioids, amphetamines, and cocaine), the withdrawal criterion of the diagnosis was measured as a syndrome, requiring at least two positive symptoms of withdrawal as defined in the DSM–IV corresponding withdrawal category. AUDADIS–IV diagnoses of abuse required a respondent to meet at least one of the four criteria defined for abuse in the 12-month period preceding the interview and not meet criteria for dependence. All drug-specific diagnoses of abuse and dependence were derived using the same algorithm and were aggregated to produce measures of any drug use disorder, any drug abuse, and any drug dependence.

The test–retest reliabilities of AUDADIS–IV alcohol and drug disorder measures were excellent, exceeding *kappa* = 0.74 for alcohol diagnoses and *kappa* = 0.79 for drug diagnoses (Canino et al., 1999; Chatterji et al., 1997; Grant et al., 1995, 2003a; Hasin et al., 1997a). The validity of the AUDADIS–IV alcohol and drug use diagnoses is well documented (Grant, 1992, 1996a,b; Grant and Harford, 1989, 1990; Harford and Grant, 1994; Hasin and Grant, 1994a,b; Hasin et al., 1996, 1997b,c; Hasin and Paykin, 1999), including in the World Health Organization/National Institutes of Health Reliability and Validity Study (Chatterji et al., 1997; Vrasti et al., 1998; Cottler et al., 1997; Pull et al., 1997; Hasin et al., 2003).

### Assessment of Other DSM–IV Psychiatric Disorders

AUDADIS–IV included modules for assessing four mood disorders (major depression, dysthymia, mania, and hypomania) and five anxiety disorders (panic disorder with agoraphobia, panic disorder without agoraphobia, social phobia, specific phobia, and generalized anxiety). As discussed elsewhere (Grant et al., 2004b), the current (last 12 months) mood and anxiety diagnoses presented in this report are defined in DSM–IV as “primary,” or independent diagnoses. In DSM–IV, the term primary is used as a shorthand to indicate those mental disorders that are not substance-induced and are not due to a general medical condition (American Psychiatric Association, 1994, p. 192). Respondents classified with disorders that were substance-induced and/or due to a general medical condition were not included in the analyses presented here. Diagnoses of major depression reported here also ruled out bereavement. All mood and anxiety disorders also satisfied the clinical significance criteria of DSM–IV by requiring distress and/or social/occupational dysfunction. The four mood disorder diagnoses were combined to create a single variable indicating the presence of any mood disorder in the past 12 months. In a similar manner, the five separate anxiety diagnoses were combined to create an anxiety disorder classification.

AUDADIS–IV also included items for the assessment of seven personality disorders (PDs): avoidant, dependent, obsessive-compulsive, paranoid, schizoid, histrionic, and antisocial personality disorder. The diagnosis of PDs requires an evaluation of an individual’s long-term patterns of functioning (American Psychiatric Association, 1994). Diagnoses of PDs made using AUDADIS–IV were made accordingly. Respondents were asked a series of personality symptom questions about how they felt or acted most of the time throughout their lives regardless of the situation or whom they were with. They were reminded on 20 occasions throughout the PD section not to include times when they were depressed, manic, anxious, drinking heavily, using medicines or drugs, or experiencing withdrawal symptoms (defined earlier in AUDADIS–IV), or times when they were physically ill. To receive a DSM–IV diagnosis, respondents needed to endorse the requisite number of DSM–IV symptom items for the particular PD and at least one positive symptom item must have caused social and/or occupational dysfunction. For this report, the seven personality disorder diagnoses were combined into a single classification reflecting the diagnosis of any of these personality disorders. The reliability and validity of AUDADIS–IV mood, anxiety, and personality disorders were fair to excellent and have been presented and reported in detail elsewhere (Canino et al., 1999; Grant et al., 1995, 2003a, 2004a,b,c, 2005; Hasin et al., 1997a).

### Alcohol and Drug Treatment

NESARC respondents who indicated a lifetime history of any alcohol consumption were asked about their use of alcohol treatment services in any 1 of 13 different treatment settings for the 12 months immediately preceding the interview and for the period of time before that. Drug users were asked a similar set of questions about drug treatment services in any 1 of 14 treatment settings. The treatment settings included 12-step programs, family or other social services agencies, alcohol or drug detoxification facilities, inpatient wards of general hospitals or community mental health programs, outpatient clinics, alcohol or drug rehabilitation programs, methadone maintenance programs (for drug treatment seekers), emergency rooms, halfway houses or therapeutic communities, crisis centers, employee assistance programs, clergy or religious counselors, private physicians or other health professionals, or any other agencies or professionals.

The structure and placement of the alcohol and drug treatment sections of AUDADIS–IV were designed to collect more reliable data that would differentiate between treatment specifically sought for alcohol and drug use disorders, even if treatment was sought for both alcohol and drugs simultaneously. AUDADIS–IV uniquely inquires about alcohol and drug treatment in separate sections of the diagnostic interview. Specifically, questions about treatment utilization for alcohol problems are asked after extensive, detailed information is obtained on alcohol consumption patterns and 40 alcohol symptom items. Similarly, drug treatment utilization questions are asked in another section of the interview preceded by detailed questions on drug use patterns and 42 drug symptom items for each of 10 specific drugs.

### Statistical Analysis

Cross-tabulations were used to calculate percent distributions, and prevalence and comorbidity rates of alcohol and drug use disorders. A series of multivariate logistic regression analyses were used to assess the strength of associations between any alcohol use disorder and a number of specific drug use disorders controlling for age, race/ethnicity, sex, education, marital status, income, region of the country, urbanicity, and current comorbid personality, mood, and anxiety disorders. Because of the complex survey design of NESARC, variance estimation procedures that assume simple random sampling cannot be employed. The stratification of the NESARC sample will result in standard errors much larger than those that would be obtained with a simple random sample of equal size. To take into account this NESARC sample design component, all standard errors and 95 percent confidence limits presented here were generated using SUDAAN (Research Triangle Institute, 2004), a software program that uses appropriate statistical techniques to adjust for sample design characteristics.

## Results

### Prevalence of DSM–IV Alcohol and Specific Drug Use Disorders: 2001–2002

The 12-month prevalence rates and population estimates of DSM–IV alcohol use disorder and specific drug use disorders in 2001–2002 are presented in Table 1. Overall, the 12-month prevalence of any alcohol use disorder was 8.46 percent. The comparable prevalence for any drug use disorder was 2.0 percent. When examined in terms of comorbidity, 7.35 percent, 0.90 percent, and 1.1 percent of the adult U.S. population had an alcohol use disorder only, a drug use disorder only or a comorbid alcohol and drug use disorder in the 12 months prior to their interview, respectively.

### Percent Distributions for Selected Demographic Characteristics and Psychiatric Disorders

Table 2 shows percent distributions for selected demographic characteristics and psychiatric disorders among three groups of respondents: (1) those with a 12-month alcohol use disorder but no 12-month drug use disorder (alcohol-only group); (2) those with a 12-month drug use disorder but no 12-month alcohol use disorder (drug-only group); (3) those with both alcohol and drug use disorders in the past 12 months (comorbid group). Group differences shown in Table 2 and discussed in this report are significant at the 0.05 level at least, and many are significant with much smaller *p*-values.

The percentage of males was significantly greater in the alcohol-only (69.4 percent) and comorbid (73.9 percent) groups than in the drug-only group (60.1 percent). There were significantly more Whites in the alcohol-only group than in the other two groups. The percent of Blacks in the alcohol-only group (8.7 percent) was significantly lower than in the drug-only (15.8 percent) group. The percentage of 18- to 29-year-olds in the comorbid (65.0 percent) group was also significantly greater than in the drug-only (47.8 percent) group, and the drug-only group (47.8 percent) had a significantly higher percentage of 18- to 29-year-olds than the alcohol-only group (38.3 percent).

The percentage of respondents who were never married decreased significantly from one group to the next in the following order: comorbid (63.2 percent), drug-only (42.2 percent), and alcohol-only (35.6 percent). Respondents in the alcohol-only (47.7 percent) and drug-only (45.0 percent) groups were significantly more likely to be married compared to the comorbid (20.2 percent) group. Furthermore, the percentage of respondents with at least some college was greatest in the alcohol-only (59.8 percent) group, compared with the drug-only (43.6 percent) and comorbid (48.4 percent) groups. Similarly, respondents in the drug-only and comorbid groups were significantly more likely to have less than a high school education and fall in the lowest income bracket than those in the alcohol-only group. Respondents in the alcohol-only group were also significantly more likely than respondents in the other two groups to be in the highest two income brackets.

There were no differences in urbanicity observed among the three groups. Respondents in the alcohol-only (29.4 percent) group were significantly more likely to live in the Midwest compared to the drug-only (20.5 percent) group, more likely to live in the South relative to the comorbid group (31.1 percent versus 24.7 percent), and less likely to live in the West compared to the drug-only and comorbid groups (21.8 percent versus 31.0 percent and 29.2 percent).

When psychopathology was examined, a consistent pattern arose among the three groups. The drug-only (44.0 percent, 27.5 percent, and 24.0 percent) and comorbid (50.8 percent, 35.3 percent, and 26.5 percent) groups were more likely to have comorbid personality, mood, and anxiety disorders compared to the alcohol-only (25.3 percent, 16.4 percent, and 15.6 percent) group. There were no significant differences in psychopathology observed between the drug-only and comorbid groups.

### Twelve-Month Prevalence of Alcohol Use Disorder Among Those With 12-Month Specific Drug Use Disorders

Table 3 presents the conditional probabilities or prevalences of 12-month alcohol use disorders among those having and not having specific drug use disorders. These are typically referred to as comorbidity rates. The prevalences of alcohol use disorders among those with drug use disorders were uniformly high, exceeding 50 percent in 20 of the 25 comparisons. The prevalence of alcohol use disorders was greatest among those with hallucinogen dependence (100 percent), followed by cocaine dependence (89.38 percent), cocaine use disorder (79.35 percent), and hallucinogen use disorder (79.16 percent). The drug use disorders with the lowest 12-month prevalences of any alcohol use disorder were sedative dependence (22.76 percent), sedative use disorder (39.76 percent), and tranquilizer dependence (43.06 percent). With the exception of sedative dependence, the prevalences of alcohol use disorders among those with a drug use disorder were significantly greater than the corresponding prevalences among those without a drug use disorder.

### Twelve-Month Prevalence of Specific Drug Use Disorders Among Those With a 12-Month Alcohol Use Disorder

Prevalences of 12-month specific drug use disorders among those with and without a 12-month alcohol use disorder are shown in Table 4. The prevalences of any drug use disorder among respondents with an alcohol use disorder were substantially lower than the corresponding prevalences of alcohol use disorders among those with drug use disorders (Table 3). Among respondents with a 12-month alcohol use disorder, the prevalence of any 12-month drug use disorder was 13.05 percent, and the most prevalent specific 12-month drug use disorders were: cannabis (9.89 percent), cocaine (2.51 percent), and opioid (2.41 percent) use disorders.

In the 25 comparisons of 12-month specific drug use, disorder prevalences among those with and without any 12-month alcohol use disorder, 23 were significantly greater for those with an alcohol use disorder compared to those without an alcohol use disorder. The two exceptions were sedative dependence, and solvent/inhalant abuse.

### Associations Between 12-Month Alcohol Use Disorder and 12-Month Specific Drug Use Disorders

Associations between past-year specific drug use disorders are shown in Table 5 in the form of odds ratios (ORs) obtained from logistic regression models. Because of a high degree of association between alcohol and drug use disorders and the sociodemographic and other psychiatric disorders shown in Table 2, the logistic models were calculated controlling for these correlates. All but three of the drug use disorders (i.e., sedative dependence, hallucinogen dependence, and solvent abuse) showed a significant association (*p* < 0.05) with any alcohol use disorder. Associations were greater for any drug dependence (OR = 9.9) than for any drug abuse (OR = 5.7).

Focusing on specific drug use disorders, the strongest associations with alcohol use disorders were observed for cocaine (OR = 19.2), followed in magnitude by hallucinogens (OR = 12.8), amphetamines (OR = 8.8), opioids (OR = 7.7), cannabis (OR = 6.8), tranquilizers (OR = 5.7), and sedatives (OR = 3.4). Interestingly, the associations were greater among individuals with dependence than abuse for opioids, amphetamines, cannabis, and cocaine, whereas the opposite was true for sedatives and tranquilizers.

### Homotypic Comorbidity and Treatment for Alcohol and Drug Use Disorders

As seen in Table 6, a very large majority of individuals with alcohol and/or drug use disorders do not seek treatment, precluding analyses for specific treatment settings. Prevalence of treatment among those with a past-year alcohol use disorder was only 6.06 percent, and the prevalence of treatment among those with any drug use disorder was significantly higher at 15.63 percent. For respondents in the comorbid group, the prevalence of treatment was 21.76 percent, which was not significantly different from those in the drug-only group. The largest group (5.29 percent) of respondents with an alcohol use disorder who sought treatment sought only alcohol treatment, whereas the largest group (12.39 percent) of those with a drug use disorder sought only drug treatment. Among the comorbid group, the prevalence of seeking alcohol-only, drug-only, and alcohol and drug treatment was 7.91 percent, 4.82 percent, and 9.04 percent, respectively, in the year preceding the interview.

## Discussion

Alcohol and drug use disorders are among the most prevalent psychiatric disorders in the United States. In 2001–2002, the 12-month prevalence of DSM–IV alcohol use disorders was 8.46 percent, representing 17.6 million adult Americans, whereas the current prevalence of drug use disorders was 2.0 percent, representing 4.2 million adult Americans. Among those with a substance use disorder, 7.35 percent and 0.90 percent were classified with alcohol use disorder only and drug use disorder only diagnoses, representing 15.3 million and 1.9 million adult Americans, respectively. The rate of comorbid alcohol and drug use disorders was 1.10 percent, representing 2.3 million adult Americans.

There was substantial variation among the three groups defined in terms of the presence or absence of current alcohol and drug use disorders. Individuals in the comorbid and drug-only groups were more likely to be young, male, never married, and of lower socioeconomic status compared with the alcohol-only group. Individuals in the drug-only group were less likely to be male, young, and never married compared with those in the comorbid group. In contrast, those in the alcohol-only group were more likely to be White, 30–64 years old, and of higher socioeconomic status relative to those in the drug-only and comorbid groups. Blacks were also overrepresented in the drug-only group when compared to those in the alcohol-only group.

The first-time availability of detailed, current alcohol and drug use disorders and their homotypic comorbidity has allowed the identification of subgroups of the population at high risk of alcohol and drug use disorders and those who are more likely to be comorbid for each of these disorders. Taken together, these results highlight the need to strengthen existing prevention and intervention efforts and to develop new programs for alcohol and drug use disorders with these observed socioeconomic differentials in mind. Moreover, these findings underscore the need for early alcohol and drug prevention programs among young, unmarried males of lower socioeconomic status, who are more likely to be comorbid for alcohol and drug use disorders, regardless of race–ethnicity.

Individuals in the drug-only and comorbid groups were more likely to have a current comorbid mood, anxiety, or personality disorder than individuals in the alcohol-only group. That psychopathology was found to be greater among individuals with drug use disorders than alcohol use disorders is consistent with recent comorbidity studies using data from NESARC (Grant et al., 2004a,b). In these studies, associations of mood, anxiety, and personality disorders with drug use disorders were much stronger than the corresponding associations with alcohol use disorders. The finding in this study that alcohol and drug use disorder comorbidity does not significantly increase the likelihood of mood, anxiety, and personality disorder comorbidity above that found among individuals with drug use disorders alone is a new finding worthy of replication and further study.

The prevalences of having an alcohol use disorder among those with a specific drug use disorder were consistently high (from 23 percent for sedative dependence to 100 percent for hallucinogen dependence) and significantly greater than the prevalences of having an alcohol use disorder among those without a drug use disorder. The only comparable numbers available from other sources come from the Burns and Teesson (2002) study using the 1997 Australian NSMHWB. For some drug categories, the NESARC conditional probabilities were similar to those from NSMHWB (e.g., sedative use disorder, U.S. 39.79 percent versus Australia 36.5 percent; stimulant use disorder, 62.84 percent versus 61.8 percent). In other comparisons, the NESARC conditional probabilities appeared to be much larger than the Australian numbers (i.e., cannabis use disorder, 57.63 percent versus 37.1 percent; opioid use disorder, 57.53 versus 31.2 percent; any drug use disorder, 55.17 percent versus 35.5 percent).

Consistent with other studies (Grant and Pickering, 1996; Ross, 1995), the prevalences of drug use disorders among those with an alcohol use disorder were considerably smaller than the prevalences of an alcohol use disorder among those with specific drug use disorders, a result, in part, attributable to the lower base rates of drug as opposed to alcohol use disorders. These prevalences ranged from 0.17 percent for solvent/inhalant abuse to 13 percent for any drug use disorder. Nonetheless, prevalences of specific drug use disorders among those with an alcohol use disorder were significantly greater than the corresponding rates among those who did not have an alcohol use disorder for all but solvent/inhalant abuse and sedative dependence. In comparison to the Australian survey, the NESARC prevalences appeared to be somewhat smaller (e.g., sedative use disorder, 0.75 percent versus 2.9 percent; stimulant use disorder, 1.22 percent versus 3.6 percent; cannabis use disorder, 9.89 percent versus 13.8 percent; any drug use disorder, 13.05 percent versus 16.8 percent), but the differences are probably not significant. Among those with an opioid use disorder, the prevalence of alcohol use disorders was 2.41 percent compared to 1.5 percent in the Australian survey. Differences between the results of this study and NSMHWB may reflect historical or cultural differences, differential availability of alcohol and specific drugs, or other economic or social factors operating in each country.

With the exception of solvent/inhalant abuse, associations between alcohol use disorders and all other specific drug use disorders were positive and significant, ranging from a low of 3.2 for sedative dependence to a high of 92.4 for cocaine dependence. The magnitudes of the associations found in this study are similar to those found in NSMHWB (i.e., sedative use disorder, 7.2 versus 9.2; stimulant use disorder, 18.5 versus 26.1; cannabis use disorder, 16.2 versus 10.5; opioid use disorder, 15.0 versus 7.2; any drug use disorder, 15.2 versus 10.1). Interestingly, associations between alcohol use disorders were not always stronger for specific drug dependence compared with specific drug abuse. Associations between alcohol use disorders were greater for abuse than dependence on sedatives and tranquilizers. In contrast, the comparable associations were greater for dependence than abuse for opioids, amphetamines, cannabis, and cocaine. Understanding why abuse and dependence on specific drugs differentially relate to alcohol use disorders may provide clues regarding the etiology of both alcohol and drug use disorders.

Similar to the results of Grant and Pickering (1996), more individuals sought treatment in the past 12 months in the drug-only group (15.63 percent) compared to the alcohol-only group (6.06 percent), whereas treatment-seeking was greatest among comorbid individuals (21.76 percent). These findings suggest the severity of alcohol and/or drug use disorders may be greater among comorbid individuals, thereby increasing help-seeking among them relative to the two noncomorbid groups examined in this study. Alternatively, the drug-only and comorbid groups may be more likely to seek alcohol and/or drug treatment because of the greater prevalence of current comorbid mood, anxiety, and personality disorders. Nonetheless, it remains unclear if increased treatment-seeking among comorbid individuals is due to severity of the substance use disorders, comorbid pathology, or the result of other factors not explored in this study. Further research should explore numerous factors influencing treatment entry, including a full array of predisposing, enabling, and need factors.

This study also found that, among those who sought some form of substance abuse treatment, the majority of individuals in the alcohol-only group sought alcohol treatment, while those in the drug-only group sought drug treatment. This was not the case for the comorbid group. Of the 21.76 percent of comorbid individuals who sought treatment in the last 12 months, 9.04 percent sought both alcohol and drug treatment, 7.91 percent only sought alcohol treatment, and 4.82 percent only sought drug treatment. The latter findings support the continuing trend toward integration of alcohol and drug treatment services, a goal which obviously has not been met. The need to integrate substance use treatment services is also underscored by recent findings from the National Survey of Substance Abuse Treatment Services (N–SSATS) (Substance Abuse and Mental Health Services Administration, 2004) which found that of the 1.1 million people in alcohol and drug treatment on a typical day in 2003, 47 percent were treated for drug and alcohol use disorders.

Perhaps one of the most interesting results in this study was the sheer number of persons with alcohol use disorders, drug use disorders, and those with alcohol and drug use disorders who were missing from the treated population. The explanation of why a clear majority of individuals with substance use disorders do not seek treatment, regardless of their comorbidity status, would require a more in-depth analysis of factors impacting on treatment not presented here. Future studies using the NESARC data promise to shed light on this unmet treatment need and will address this important issue by examining reasons why individuals with alcohol and/or drug use disorders did not seek treatment.

In conclusion, this study has contributed to our knowledge of the prevalence, comorbidity, treatment-seeking, and risk factors of alcohol and drug use disorders. The findings from this study provide information that can be used to improve prevention and intervention programs and increase our knowledge of the development of alcohol and drug use disorders. Further work in many directions is indicated by the results of this study, including the factors giving rise to the associations between alcohol and drug use disorders, the treatment implications of these disorders when comorbid, and the impact of other comorbid psychiatric disorders on the development of alcohol use disorders, drug use disorders, and their comorbidity.

## Figures and Tables

**Table 1:** Twelve-Month Prevalences and Population Estimates of DSM–IV Alcohol and Drug Use Disorders

Disorder	%	S.E.	Population Estimate^a^
Alcohol use disorder	8.46	0.24	17580
Alcohol use disorder only	7.35	0.22	15285
Any drug use disorder only	0.90	0.07	1864
Alcohol use disorder and any drug use disorder	1.10	0.07	2295
Any drug use disorder	2.00	0.10	4159
Any drug abuse	1.37	0.08	2858
Any drug dependence	0.63	0.05	1301
Sedative use disorder	0.16	0.02	333
Sedative abuse	0.09	0.02	193
Sedative dependence	0.07	0.01	140
Tranquilizer use disorder	0.13	0.02	260
Tranquilizer abuse	0.08	0.02	163
Tranquilizer dependence	0.05	0.01	977
Opioid use disorder	0.35	0.05	737
Opioid abuse	0.24	0.04	500
Opioid dependence	0.11	0.02	236
Amphetamine use disorder	0.16	0.03	342
Amphetamine abuse	0.09	0.02	196
Amphetamine dependence	0.07	0.02	146
Hallucinogen use disorder	0.14	0.02	291
Hallucinogen abuse	0.12	0.02	259
Hallucinogen dependence	0.02	0.01	32
Cannabis use disorder	1.45	0.08	3016
Cannabis abuse	1.13	0.06	2342
Cannabis dependence	0.32	0.04	674
Cocaine use disorder	0.27	0.03	557
Cocaine abuse	0.13	0.02	277
Cocaine dependence	0.13	0.02	280
Solvent/inhalant abuse^b^	0.02	0.01	49

a Population estimates are in thousands.

b There were no diagnoses of solvent/inhalant dependence.

**Table 2:** Percent Distributions of Selected Demographic Characteristics and DSM–IV Psychiatric Disorders Among Respondents With and Without Alcohol and/or Any Drug Use Disorders

Demographic Characteristic/Psychiatric Disorder	Alcohol Use Disorder Only (*n* = 2903)		Any Drug Use Disorder Only (*n* = 353)		Alcohol and Any Drug Use Disorder (*n* = 424)		No Alcohol or Drug Use Disorder (*n* = 39,413)	
			
	%	S.E.	%	S.E.	%	S.E.	%	S.E.
Sex								
Male	69.4^a^,^c^	1.04	60.1^b^,^c^	2.97	73.9^c^	2.64	45.7	0.32
Female	30.6^a^,^c^	1.04	39.9^b^,^c^	2.97	26.1^c^	2.64	54.3	0.32
Race–ethnicity								
White	75.8^a^,^b^,^c^	1.85	68.5	2.90	68.5	3.06	70.6	1.61
Black	8.7^a^,^c^	0.67	15.8^c^	2.14	11.5	1.83	11.2	0.65
Native American	2.5	1.24	3.3	1.09	6.8^c^	1.70	2.0	0.15
Asian/Pacific Islander	2.3^c^	0.48	3.6	1.61	2.6	0.99	4.6	0.56
Hispanic	10.8	1.59	8.9^c^	1.88	11.0	1.82	11.7	1.23
Age (years)								
18–29	38.3^a^,^b^,^c^	1.18	47.8^b^,^c^	3.30	65.0^c^	3.02	19.7	0.37
30–44	37.0^b^,^c^	1.09	33.8	3.19	25.9	2.63	30.4	0.33
45–64	21.6^a^,^b^,^c^	0.98	15.9^b^,^c^	2.31	9.1^c^	1.85	32.3	0.32
>65	3.2^b^,^c^	0.34	2.6^b^,^c^	0.78	0.1^c^	0.07	17.6	0.37
Marital status								
Married/living as if married	47.7^b^,^c^	1.08	45.0^b^,^c^	3.14	20.2^c^	2.21	63.4	0.50
Widowed/separated/divorced	16.7	0.85	12.8^c^	1.96	16.6	2.18	17.6	0.24
Never married	35.6^a^,^b^,^c^	1.21	42.2^b^,^c^	3.04	63.2^c^	2.80	19.0	0.49
Education level								
Less than high school	12.2^a^,^b^,^c^	0.98	18.3	2.51	18.2	2.67	15.9	0.49
High school diploma/GED	27.9^a^	1.09	38.1^c^	3.29	33.3	2.77	29.3	0.56
Some college or higher	59.8^a^,^b^,^c^	1.32	43.6^c^	2.93	48.4^c^	3.01	54.8	0.63
Personal income (US$)								
0–19,999	39.3^a^,^b^,^c^	1.22	66.1^c^	3.31	65.4^c^	2.89	47.5	0.59
20,000–34,999	25.8^a^,^c^	1.06	18.7	2.69	23.1	2.45	22.4	0.36
35,000–69,999	25.5^a^,^b^,^c^	1.00	11.3^c^	2.13	9.7^c^	1.61	21.9	0.40
>70,000	9.4^a^,^b^	0.71	4.0^c^	1.68	1.8^c^	0.72	8.2	0.38
Urbanicity								
Urban	79.6	1.83	84.3	2.60	78.4	3.09	80.3	1.62
Rural	20.4	1.83	15.7	2.60	21.6	3.09	19.7	1.62
Geographic region								
Northeast	17.8	2.98	21.1	4.23	20.5	4.21	19.8	3.46
Midwest	29.4^a^,^c^	3.30	20.5	4.16	25.7	3.72	22.6	3.19
South	31.1^b^,^c^	3.08	27.5^c^	4.03	24.7^c^	3.67	35.8	3.31
West	21.8^a^,^b^	3.33	31.0^c^	5.83	29.2	4.30	21.8	3.54
Any personality disorder^d^								
Yes	25.3^a^,^b^,^c^	1.00	44.0^c^	3.45	50.8^c^	3.05	13.2	0.33
No	74.7^a^,^b^,^c^	1.00	56.0^c^	3.45	49.2^c^	3.05	86.8	0.33
Any past-year independent mood disorder								
Yes	16.4^a^,^b^,^c^	0.81	27.5^c^	2.58	35.3^c^	3.32	8.1	0.21
No	83.6^a^,^b^,^c^	0.81	72.5^c^	2.58	64.7^c^	3.32	91.9	0.21
Any past-year independent anxiety disorder								
Yes	15.6^a^,^b^,^c^	0.86	24.0^c^	2.66	26.5^c^	2.82	10.4	0.32
No	84.4^a^,^b^,^c^	0.86	76.0^c^	2.66	73.5^c^	2.82	89.6	0.32

a Prevalence is significantly (*p* < 0.05) different from “any drug use disorder only” prevalence.

b Prevalence is significantly (*p* < 0.05) different from “alcohol and any drug use disorder” prevalence.

c Prevalence is significantly (*p* < 0.05) different from “no alcohol or drug use disorder” prevalence.

d Personality disorders assessed only on a lifetime basis.

**Table 3:** Twelve-Month Prevalence of DSM–IV Alcohol Use Disorders Among Respondents With and Without 12-Month Drug Use Disorders

Drug Use Disorder	Prevalence of Alcohol Use Disorder Among Respondents			

	With Drug Use Disorder		Without Drug Use Disorder	

	%	S.E.	%	S.E.
Any drug use disorder	55.17^a^	2.29	7.50	0.22
Any drug abuse	49.47^a^	2.71	7.88	0.23
Any drug dependence	67.69^a^	4.02	8.08	0.23
Sedative use disorder	39.79^a^	7.03	8.41	0.24
Sedative abuse	52.15^a^	9.95	8.42	0.24
Sedative dependence	22.76	8.21	8.45	0.24
Tranquilizer use disorder	57.90^a^	8.35	8.40	0.24
Tranquilizer abuse	66.16^a^	10.40	8.41	0.24
Tranquilizer dependence	43.06^c^	12.25	8.44	0.24
Opioid use disorder	57.53^a^	4.76	8.28	0.23
Opioid abuse	49.77^a^	6.45	8.36	0.24
Opioid dependence	73.96^a^	6.54	8.38	0.24
Amphetamine use disorder	62.84^a^	7.59	8.37	0.24
Amphetamine abuse	51.83^b^	10.00	8.42	0.24
Amphetamine dependence	77.64^a^	9.29	8.41	0.24
Hallucinogen use disorder	79.16^a^	6.78	8.36	0.24
Hallucinogen abuse	76.61^a^	7.44	8.37	0.24
Hallucinogen dependence	100.00^a^	0.00	8.44	0.24
Cannabis use disorder	57.63^a^	2.81	7.73	0.22
Cannabis abuse	54.69^a^	2.92	7.93	0.23
Cannabis dependence	67.86^a^	6.44	8.26	0.24
Cocaine use disorder	79.35^a^	4.56	8.27	0.24
Cocaine abuse	69.23^a^	7.57	8.38	0.24
Cocaine dependence	89.38^a^	4.25	8.35	0.24
Solvent/inhalant abuse	59.94^d^	22.36	8.44	0.24

a *p* < 0.0001.

b *p* < 0.001.

c *p* < 0.01.

d *p* < 0.05.

**Table 4:** Twelve-Month Prevalence of DSM–IV Drug Use Disorders Among Respondents With and Without 12-Month Alcohol Use Disorders

Drug Use Disorder	Prevalence of Drug Use Disorder Among Respondents			

	With Alcohol Use Disorder		Without Alcohol Use Disorder	

	%	S.E.	%	S.E.
Any drug use disorder	13.05^a^	0.74	0.98	0.07
Any drug abuse	8.04^a^	0.58	0.76	0.06
Any drug dependence	5.01^a^	0.46	0.22	0.03
Sedative use disorder	0.75^b^	0.17	0.11	0.02
Sedative abuse	0.57^c^	0.15	0.05	0.02
Sedative dependence	0.18	0.07	0.06	0.01
Tranquilizer use disorder	0.85^b^	0.20	0.06	0.01
Tranquilizer abuse	0.61^c^	0.18	0.03	0.01
Tranquilizer dependence	0.24^d^	0.08	0.03	0.01
Opioid use disorder	2.41^a^	0.37	0.16	0.03
Opioid abuse	1.42^a^	0.28	0.13	0.02
Opioid dependence	0.99^b^	0.24	0.03	0.01
Amphetamine use disorder	1.22^a^	0.21	0.07	0.02
Amphetamine abuse	0.58^b^	0.15	0.05	0.02
Amphetamine dependence	0.64^b^	0.16	0.02	0.01
Hallucinogen use disorder	1.31^a^	0.23	0.03	0.01
Hallucinogen abuse	1.13^a^	0.22	0.03	0.01
Hallucinogen dependence	0.18^d^	0.08	0.00	0.00
Cannabis use disorder	9.89^a^	0.60	0.67	0.06
Cannabis abuse	7.29^a^	0.51	0.56	0.05
Cannabis dependence	2.60^a^	0.33	0.11	0.03
Cocaine use disorder	2.51^a^	0.35	0.06	0.01
Cocaine abuse	1.09^a^	0.23	0.04	0.01
Cocaine dependence	1.42^a^	0.27	0.02	0.01
Solvent/inhalant abuse	0.17	0.10	0.01	0.01

a *p* < 0.0001.

b *p* < 0.001.

c *p* < 0.01.

d *p* < 0.05.

**Table 5:** Adjusted^a^ Odds Ratios of 12-Month DSM–IV Alcohol Use Disorders and 12-Month Specific Drug Use Disorders

Drug Use Disorder	OR	(95% CI)^b^
Any drug use disorder	7.4	(6.00–9.03)
Any drug abuse	5.7	(4.49–7.30)
Any drug dependence	9.9	(6.47–15.01)
Sedative use disorder	3.4	(1.89–6.29)
Sedative abuse	5.3	(2.44–11.31)
Sedative dependence	1.8	(0.65–4.93)
Tranquilizer use disorder	5.7	(2.55–12.69)
Tranquilizer abuse	7.1	(2.52–20.17)
Tranquilizer dependence	4.0	(1.30–12.16)
Opioid use disorder	7.7	(5.18–11.41)
Opioid abuse	6.1	(3.76–9.91)
Opioid dependence	12.9	(6.18–26.89)
Amphetamine use disorder	8.8	(4.24–18.29)
Amphetamine abuse	5.2	(2.14–12.59)
Amphetamine dependence	20.3	(6.18–66.94)
Hallucinogen use disorder	12.8	(5.18–31.49)
Hallucinogen abuse	10.7	(4.24–26.80)
Hallucinogen dependence		—c
Cannabis use disorder	6.8	(5.36–8.75)
Cannabis abuse	6.2	(4.80–7.90)
Cannabis dependence	7.3	(3.92–13.72)
Cocaine use disorder	19.2	(10.71–34.56)
Cocaine abuse	10.5	(4.85–22.56)
Cocaine dependence	43.0	(17.83–103.49)
Solvent/inhalant abuse	3.6	(0.52–25.82)

a Odds ratios adjusted for sociodemographic factors, any personality disorder, any 12-month independent mood disorder, and any independent anxiety disorder.

b 95% CI = 95% confidence interval.

c Unable to calculate OR because all respondents with hallucinogen dependence also had an alcohol use disorder.

**Table 6:** Twelve-Month Prevalence of Alcohol and/or Drug Treatment Among Respondents With DSM–IV Alcohol and/or Drug Use Disorders

Treatment Type	Alcohol Use Disorder Only		Any Drug Use Disorder Only		Alcohol and Any Drug Use Disorder	
		
	%	S.E.	%	S.E.	%	S.E.
Alcohol treatment only (*n* = 196)	5.29^a^	0.51	1.85^b^	1.08	7.91	1.43
Drug treatment only (*n* = 64)	0.39^a^,^b^	0.16	12.39^b^	1.98	4.82	1.36
Alcohol and drug treatment (*n* = 59)	0.38^b^	0.11	1.39^b^	0.58	9.04	1.64
Alcohol and/or drug treatment (*n* = 319)	6.06^a^,^b^	0.59	15.63	2.39	21.76	2.53

a Percent is significantly (*p* < 0.05) different from “any drug use disorder only” percent.

b Percent is significantly (*p* < 0.05) different from “alcohol and any drug use disorder” percent.
